# Draft genome sequences of 21 *Pedobacter* strains isolated from amphibian specimens

**DOI:** 10.1128/mra.01185-23

**Published:** 2024-02-27

**Authors:** Celine M. Zumkeller, Molly C. Bletz, Andolalao Rakotoarison, Joana Sabino-Pinto, Silke Reiter, Marius Spohn, Oliver Schwengers, Alexander Goesmann, Miguel Vences, Sanja Mihajlovic, Till F. Schäberle

**Affiliations:** 1 Institute for Insect Biotechnology, Justus-Liebig-University Giessen, Giessen, Germany; 2 Branch for Bioresources, Fraunhofer Institute for Molecular Biology and Applied Ecology (IME), Giessen, Germany; 3 Department of Environmental Conservation, University of Massachusetts Amherst, Amherst, Massachusetts, USA; 4 Mention Environnement, Université de l’Itasy, Faliarivo Ambohidanerana, Soavinandriana Itasy, Madagascar; 5 Groningen Institute for Evolutionary Life Sciences, University of Groningen, Groningen, The Netherlands; 6 Bioinformatics and Systems Biology, Justus-Liebig-University Giessen, Giessen, Germany; 7 German Center for Infection Research (DZIF), Partner Site Giessen-Marburg-Langen, Giessen, Germany; 8 Technische Universität Braunschweig, Zoological Institute, Braunschweig, Germany; California State University, San Marcos, California, USA

**Keywords:** *Pedobacter*, amphibians, Madagascar, Germany, beta-lactamases, natural products

## Abstract

The genomes of 21 *Pedobacter* strains isolated from the European salamander *Salamandra salamandra* and different Madagascan frog species were sequenced using Illumina sequencing. Here, we report their draft genome sequences (~4.7–7.2 Mbp in size) to allow comparative genomics and taxonomic assignment of these strains.

## ANNOUNCEMENT

The bacterial genus *Pedobacter* is widely distributed in many habitats and associated with macroorganisms, including amphibians (1, 2). *Pedobacter* has been identified as part of their cutaneous microbiome (3–5) and has been found to inhibit the growth of pathogenic fungi (3, 4). Certain strains are multidrug resistant and are not susceptible to beta-lactams, colistin, aminoglycosides, and ciprofloxacin (5). This phenotype is supported by a high and diverse number of antibiotic resistance genes detected in the genomes (6). Aimed at characterizing new *Pedobacter* strains, bacteria were isolated from skin swabs of salamanders and frogs from Germany and Madagascar.

For sampling and cultivation of strains with DE and EXT identifiers, see Bletz et al. (4). Briefly, amphibians, captured with clean nitrile gloves, were placed in sterile bags and rinsed with 50 mL of sterilized water before being swabbed. Swabs were stored in Tryptic Soy Yeast Extract + 20% glycerol and kept on ice before transferring to a −20°C freezer. FhG111542 and FhG11526 were isolated as follows: Salamander specimens were gently washed with sterile tap water, and bacteria were collected using sterile cotton swabs. Swabs were stored in sterile tap water at ambient temperature and processed on the same day. To release bacteria, swabs were vortexed, and the resulting bacterial suspensions were plated on agar plates (R2A: HiMedia Laboratories GmbH; Product No.: M1687 and 10% TSB: Thermo Fisher Scientific Inc.; Product No.: CM0129) and incubated at room temperature or 4°C for several days. Colonies were selected based on morphology and subcultured to obtain pure cultures. For sequencing, strains were grown aerobically in NB-medium (0.5% peptone, 0.3% malt extract, and 0.5% NaCl) at 18°C for 24–72 hours. Cell pellets were resuspended in ATL buffer (Qiagen) containing RNAse A. ZR BashingBead Lysis Tubes (Zymo Research) were used for cell disruption. DNA was isolated using QIAmp 96 DNA QIAcube HT Kits with the addition of proteinase K (Qiagen). Libraries for short-read sequencing were prepared using the Illumina DNA Prep Tagmentation Kit with 500 ng DNA input and five cycles indexing PCR. Library quality was evaluated (Agilent 2100 Bioanalyzer) and sequenced on an Illumina NovaSeq using a NovaSeq 6000 SP v1 Sequencing Kit with 2 × 150 bp read length and a depth of 4.0–5.0 million reads per sample. Unless otherwise stated, software tools were run with default settings for sequence processing and analysis. The sequence data were demultiplexed (Illumina bcl2fastq, v2.19.0.316), quality checked (Fastp, v0.20.1), and visualized (MultiQC, v1.7). Paired-end reads were quality filtered [Fastp (7) v0.20.1, additional 53 parameter: “--detect_adapter_for_pe --cut_by_quality5 --cut_by_quality3 --low_complexity_filter -−54 length_required 21 --correction”], assembled [Unicycler (8) v0.4.8], and quality checked [CheckM2 (9) v1.0.18]. Taxonomical ranks were established using the Type Strain Genome Server (10) and GTDB (11).

It has been proposed that all *Pedobacter* genus members commonly encode beta-lactamases (6). Thus, we predicted resistosomes by RGI and extracted putative beta-lactamases using the Comprehensive Antibiotic Resistance Database (12). These sequences were used to construct a sequence similarity network (SSN) using Enzyme function initiative-enzyme similarity tool (EFI-EST) (13) (restricted to 200–440 amino acids, alignment score threshold: 20). The SSN revealed 118 putative beta-lactamases (per strain on average 5.6 ± 2.5) clustering to 89 reference enzymes. Based on the SSN, the candidate beta-lactamases were assigned to class C (*n* = 86), class B3 (*n* = 13), class A (*n* = 11), and class B1/B2 (*n* = 3). Notably, we identified five putative beta-lactamases clustering to class D references, which have not yet been reported in *Pedobacter*. Our analysis indicates the presence of at least two beta-lactamases, suggesting a general potential for beta-lactam inactivation in this genus.

**TABLE 1:** Sequencing characteristics (light gray entries) and beta-lactamase distribution (dark gray entries) of the 21 *Pedobacter* isolates

Isolation			Sequence details						NCBI information			No. of detected genes/beta-lactamase class				
Isolate (ID)	Isolation source	Sampling location (Lat., Long.)	Closest type strain (TYGS)	No. of raw reads	Contig no.	N50^*a*^ (bp)	GC (%)	Assembly size (bp)	GenBank accession	SRA accession	Biosample accession (SAMN)	A	B1B2	B3	C	D
DE_ 0159	*Salamandra salamandra*	Kottenforst, Germany	*P. frigiditerrae* RP-1-13	2.74E + 07	13	2.16E + 06	35	4.83E + 06	JAVTSV000000000	SRR26200159	37505411	0	0	1	3	0
DE_ 0302	*Salamandra salamandra*	Harz, Germany	*P. miscanthi* RS10	2.66E + 07	137	2.88E + 05	39	7.00E + 06	JAVTSU000000000	SRR26200158	37505412	1	0	1	4	0
DE_ 0380	*Salamandra salamandra*	Solling, Germany	*P. gandavensis* LMG 31462T	3.02E + 07	45	6.96E + 05	40	5.92E + 06	JAVTST000000000	SRR26200147	37505413	1	0	0	4	1
DE_ 0385	*Salamandra salamandra*	Solling, Germany	*P. frigiditerrae* RP-1–13	2.24E + 07	4	2.62E + 06	35	4.85E + 06	JAVTSS000000000	SRR26200145	37505414	0	0	1	3	0
DE_ 0392	*Salamandra salamandra*	Solling, Germany	*P. agri* DSM 19486	2.66E + 07	83	2.42E + 05	37	5.19E + 06	JAVTSR000000000	SRR26200144	37505415	0	0	1	3	0
DE_ 0410	*Salamandra salamandra*	Solling, Germany	*P. nutrimenti* DSM 27372	2.19E + 07	64	4.25E + 05	41	6.95E + 06	JAVTSQ000000000	SRR26200143	37505416	1	0	0	5	2
DE_ 0497	*Salamandra salamandra*	Solling, Germany	*P. frigoris* KACC 21154	2.41E + 07	33	7.15E + 05	40	5.13E + 06	JAVTSP000000000	SRR26200142	37505417	0	0	0	2	0
DE_ 0550	*Salamandra salamandra*	Harz, Germany	*P. nototheniae* 36B243T	2.09E + 07	54	6.30E + 05	37	4.90E + 06	JAVTSO000000000	SRR26200141	37505418	0	1	1	7	0
DE_ 0801	*Salamandra salamandra*	Solling, Germany	*P. psychrodurus* RP-3-21	2.51E + 07	214	1.20E + 05	40	7.20E + 06	JAVTSN000000000	SRR26200140	37505419	1	0	1	7	0
DE_ 0989	*Salamandra salamandra*	Eifel, Germany	*P. antarcticus* DSM 11725	3.01E + 07	50	3.89E + 05	41	4.86E + 06	JAVTSM000000000	SRR26200139	37505420	1	0	1	8	1
MADA_ 173	*Boophis williamsi*	Ankaratra, Madagascar (−19.3463, 47.27705)	*P. antarcticus* DSM 11725	1.86E + 07	49	2.63E + 05	41	5.05E + 06	JAVTSL000000000	SRR26200157	37505421	1	0	1	7	1
MADA_ 278	*Mantidactylus aff. curtus 19*	Ankaratra, Madagascar (−19.3463, 47.27705)	*P. gandavensis* LMG 31462T	2.99E + 07	73	4.19E + 05	40	5.26E + 06	JAVTSK000000000	SRR26200156	37505422	2	0	0	4	0
MADA_ 852	*Aglyptodactylus madagascariensis*	Andasibe, Madagascar (−18.9328, 48.41312)	*P. aquatilis* CECT 7114	2.65E + 07	30	5.43E + 05	39	4.94E + 06	JAVTSJ000000000	SRR26200155	37505423	1	0	1	3	0
MADA_1817	*Ptychadena mascareniensis*	Andasibe, Madagascar (−18.9328, 48.41312)	*P. agri* DSM 19486	2.32E + 07	35	1.53E + 06	38	5.00E + 06	JAVTSI000000000	SRR26200154	37505424	0	0	1	3	0
MADA_1818	*Ptychadena mascareniensis*	Andasibe, Madagascar (−18.9328, 48.41312)	*P. agri* DSM 19486	3.12E + 07	40	4.58E + 05	38	5.03E + 06	JAVTSH000000000	SRR26200153	37505425	0	0	1	3	0
MADA_2501	*Boophis goudoti*	Ankaratra, Madagascar (−19.3463, 47.27705)	*P. aquatilis* CECT 7114	3.65E + 07	17	7.73E + 05	36	4.68E + 06	JAVTSG000000000	SRR26200152	37505426	0	1	1	3	0
MADA_2608	*Mantella aurantiaca*	Breeding center, Madagascar	*P. nyackensis* DSM 19625	2.62E + 07	51	7.17E + 05	39	6.23E + 06	JAVTSF000000000	SRR26200151	37505427	1	0	0	3	0
MADA_3128	*Spinomantis aglavei*	Andasibe, Madagascar (−18.9328, 48.41312)	*P. frigidisoli* RP-3-11	1.94E + 07	50	6.98E + 05	41	5.18E + 06	JAVTSE000000000	SRR26200150	37505428	0	0	0	3	0
MADA_3506	*Boophis goudoti*	Ankaratra, Madagascar (−19.3463, 47.27705)	*P. aquatilis* CECT 7114	2.41E + 07	29	5.45E + 05	36	5.23E + 06	JAVTSD000000000	SRR26200149	37505429	0	0	1	6	0
S10_4.1	*Salamandra*	Schiffenberg Forest, Germany	*P. heparinus* DSM 2366	2.26E + 07	86	2.59E + 05	42	5.74E + 06	JAVTSC000000000	SRR26200148	37505430	0	1	0	3	0
S8_12.1	*Salamandra*	Schiffenberg Forest, Germany	*P. gandavensis* LMG 31462T	1.49E + 07	60	3.77E + 05	41	5.72E + 06	JAVTSB000000000	SRR26200146	37505431	1	0	0	2	0

^*a*^ The N50 value denotes the sequence length of the shortest contig at 50% of the total assembly length.

## Data Availability

The whole-genome shotgun project has been deposited at GenBank under the BioProject accession number PRJNA1019955. The draft genome sequences have been deposited at GenBank under the accession numbers in Table 1. The BioProject metadata include all relevant information on the collection site, date, and host. If applicable, the exact geographic sampling locations are included in Table 1. Whenever exact coordinates are not included, multiple individuals were sampled, leading to the description of overall sampling areas (Table 1).
